# Timing Is Important: Unmanned Aircraft vs. Satellite Imagery in Plant Invasion Monitoring

**DOI:** 10.3389/fpls.2017.00887

**Published:** 2017-05-31

**Authors:** Jana Müllerová, Josef Brůna, Tomáš Bartaloš, Petr Dvořák, Michaela Vítková, Petr Pyšek

**Affiliations:** ^1^Institute of Botany, The Czech Academy of SciencesPrůhonice, Czechia; ^2^Faculty of Science, Institute for Environmental Studies, Charles UniversityPrague, Czechia; ^3^GISAT Ltd.Prague, Czechia; ^4^Institute for Aerospace Engineering, Brno University of TechnologyBrno, Czechia; ^5^Department of Ecology, Faculty of Science, Charles UniversityPrague, Czechia

**Keywords:** alien species, giant hogweed, knotweed, plant phenology, remote sensing detection, UAV

## Abstract

The rapid spread of invasive plants makes their management increasingly difficult. Remote sensing offers a means of fast and efficient monitoring, but still the optimal methodologies remain to be defined. The seasonal dynamics and spectral characteristics of the target invasive species are important factors, since, at certain time of the vegetation season (e.g., at flowering or senescing), plants are often more distinct (or more visible beneath the canopy). Our aim was to establish fast, repeatable and a cost-efficient, computer-assisted method applicable over larger areas, to reduce the costs of extensive field campaigns. To achieve this goal, we examined how the timing of monitoring affects the detection of noxious plant invaders in Central Europe, using two model herbaceous species with markedly different phenological, structural, and spectral characteristics. They are giant hogweed (*Heracleum mantegazzianum*), a species with very distinct flowering phase, and the less distinct knotweeds (*Fallopia japonica, F. sachalinensis*, and their hybrid *F*. × *bohemica*). The variety of data generated, such as imagery from purposely-designed, unmanned aircraft vehicle (UAV), and VHR satellite, and aerial color orthophotos enabled us to assess the effects of spectral, spatial, and temporal resolution (i.e., the target species' phenological state) for successful recognition. The demands for both spatial and spectral resolution depended largely on the target plant species. In the case that a species was sampled at the most distinct phenological phase, high accuracy was achieved even with lower spectral resolution of our low-cost UAV. This demonstrates that proper timing can to some extent compensate for the lower spectral resolution. The results of our study could serve as a basis for identifying priorities for management, targeted at localities with the greatest risk of invasive species' spread and, once eradicated, to monitor over time any return. The best mapping strategy should reflect morphological and structural features of the target plant and choose appropriate spatial, spectral, and temporal resolution. The UAV enables flexible data acquisition for required time periods at low cost and is, therefore, well-suited for targeted monitoring; while satellite imagery provides the best solution for larger areas. Nonetheless, users must be aware of their limits.

## Introduction

Invasive species are considered among the major drivers of global change and are threats to biodiversity, ecosystem services, the economy, and human health (Pyšek and Richardson, 2010; Vilà et al., 2010). Early monitoring and rapid action at the operational level are therefore needed to mitigate the consequences of plant invasions (Kaiser and Burnett, 2010). Thanks to the availability of high spatial-resolution data, remote sensing (RS) is being increasingly used to study and model the spread of invasive plant species (Pergl et al., 2011; Somers and Asner, 2013; Rocchini et al., 2015), their phenology (Ge et al., 2006), and impacts (Asner et al., 2008). Most of the RS studies focus on invasive trees and shrubs (e.g., Frazier and Wang, 2011; Masocha and Skidmore, 2011), but the accurate detection of herbaceous plant species is challenging owing to their smaller size and patchiness. In this sense, invasive plant species are easier to handle compared to native ones since they usually grow vigorously, produce a great amount of biomass, and dominate the canopy, often forming extensive monospecific stands (Maheu-Giroux and de Blois, 2004; Huang and Asner, 2009; Müllerová et al., 2013). There continues to be a need for data of sufficient quality and resolution, especially where the target species is less distinct from other vegetation, or covered by it.

It has been shown that the timing of data acquisition plays an important role (Laba et al., 2005) since plants are often more distinct from the surrounding vegetation at certain times of the vegetation season (Andrew and Ustin, 2008; Rocchini et al., 2015), mostly during flowering (Müllerová et al., 2005, 2013; Andrew and Ustin, 2006; Ge et al., 2006) or leaf coloring (Shouse et al., 2013). Using a change detection approach, some species can be recognized by the differences in their life cycle compared to the background vegetation (Peterson, 2005; McEwan et al., 2009). Such adaptation is quite common in plant invasions, since the mismatch of phenology between native and exotic species can provide benefit to invaders outcompeting the natives by using the time window available via extending or shifting the growing season (Wolkovich and Cleland, 2011; Fridley, 2012; Gioria et al., 2016). Phenological differences influencing the RS detection include also the seasonal development of broad-leaved tree canopies, because the target species in the understory may be detectable only before emergence and after the leaf senescence of the canopy vegetation (Resasco et al., 2007; Wilfong et al., 2009). Thus, the seasonal dynamics and spectral behavior of the target species and their surrounding constitute important parameters in the RS detection process.

To study the role of phenology on RS detection of invasive species, it is necessary to acquire data at certain phenological phases, as well as with suitable spatial and spectral resolution. Freely available satellite data such as MODIS, Landsat or Sentinel do not provide appropriate spatial resolution for a highly heterogeneous Central European landscape where the occurrence of invasive plant populations is rather patchy. Very high resolution (VHR) satellites such as Pleiades, QuickBird, Ikonos, or WorldView are costly to acquire, and in all cases the acquisition of suitable data is constrained by clouds and the regular trajectory of the satellite. Aerial campaigns offer some flexibility, but they must still be planned in advance, with uncertainty in weather forecasting being a factor, and they are also costly. If relying on the archives of aerial imagery, the choice of phenology phases is limited and spectral resolution is usually low. New means such as unmanned aircraft vehicles (UAV) provide high flexibility, low price, and easy deployment (Watts et al., 2012), but rather low spectral resolution (RGB + modified NIR bands with modified consumer cameras if cheap solution is considered), and due to geometric and radiometric irregularities they require complex processing (Rango et al., 2009; Laliberte et al., 2010; Salamí et al., 2014; Müllerová et al., 2017). At the same time, the large amount of data collected by UAVs brings about the need for automatic processing. Legal constraints limiting use of UAVs only to unpopulated areas further reduce their applicability (Watts et al., 2012). This is especially problematic for invasive species that tend to prefer urban areas (Pyšek, 1998; Kowarik, 2008). Moreover, extremely high detail of UAV data generates problems with precise location of validation data due to the limits of available GNSS instruments (especially in forested or hilly environments). Still, despite these limits, the UAV technology holds great potential for invasive species assessment and monitoring (Calviño-Cancela et al., 2014; Michez et al., 2016; Müllerová et al., 2016, 2017; Table 1).

**Table 1:** Advantages and constraints of different types of remote sensing imagery in invasive species monitoring.

	**NASA and ESA satellites**	**VHR satellites**	**Aerial**	**UAV**
Timing (flexibility)	No flexibility, but extensive archive	Low	Medium	**High**
Resolution	Down to 10 m	Down to 0.3–0.4 m	0.1–0.5 m	**up to 1 cm**
Financial costs of imagery	**No**	High	High	**Low**
Acquisition	**Easy—available for download**	**Easy—commercial order**	**Easy—commercial order**	Some expertise needed
Pre-processing	**Standardized**	**Standardized**	**Standardized**	Not standardized, complex
Weather constraints—cloudy sky	Impossible	Impossible	Impossible	**Only possible in case of high clouds and no rain**
Weather constraints—wind	**No influence**	**No influence**	Strong wind is problematic	Very problematic
Legal constraints	**No**	**No**	Few	Many constraints (e.g., in urban areas, private land, commercial zones, around airports…)
Data volume	**Moderate**	High	High	High/very high
Spectral resolution	**High**	**High**	Low (medium)	Medium (depend on camera)
Temporal resolution	**High**	Moderate	Low	**High**

*The best options are highlighted in bold*.

To demonstrate the role of timing and resolution for the RS detection of invasive plants, we chose two model herbaceous species with markedly different phenological, structural, and spectral characteristics. They are the giant hogweed (*Heracleum mantegazzianum*), a species with a very distinct flowering phase, during which it forms large white circular inflorescences, and knotweeds (*Fallopia japonica, F. sachalinensis*, and their hybrid *F*. × *bohemica*), lacking such distinct features. High flexibility of the data acquisition provided by small UAV (in our case a custom-built one producing imagery of 5 cm spatial and multispectral resolution of RGB + modified NIR) enabled us to study the role of phenology. The results were compared with those of multispectral satellite imagery of 50 cm resolution using several approaches (pixel- and object-based) and data from different parts of the vegetation season to find a repeatable, efficient, and low-cost monitoring strategy (in terms of data source, processing approach and target species' phenological stage). Our aims were to: (a) assess the role of timing and both the spectral and spatial resolution in detection of the two structurally and phenologically different invasive species, (b) define the optimal phenological stage (time window) and data resolution, and (c) describe the computer-assisted workflow of the image-processing to achieve the highest detection rate. Our recommended methodology, operational in environmental monitoring and applicable over large areas, can be used for nature conservation, with the potential to reduce the costs of extensive field campaigns.

## Methods

### Study species

To study the different aspects important for their recognition, we chose model herbaceous invasive species differing in plant architecture and spectral dynamics: giant hogweed and knotweeds (Table 2). These plants are among the most serious plant invaders in Europe (DAISIE, 2012).

**Table 2:** Remote sensing detection of the two target species—their important characteristics, imagery required for the analysis and the best processing approach.

**Species**		***Heracleum mantegazzianum* (giant hogweed)**	***Fallopia* sp. (knotweeds)**
Foliage structure		Large, deeply incised leaves	Variable leaves, forming dense stands
Inflorescence		Large, distinct	Small, insignificant
Type of habitat infested		Unmanaged grasslands, abandoned land, riverbanks, sparse forest and field edges, ruderal habitats	Riverbanks, unmanaged grasslands, disturbed sites, sparse forests, urban areas
Optimal phenological stage for RS detection		Peak of flowering	Senescence
Optimal period of the data acquisition (CR)		2nd half of June—1st half of July	End of October, beginning of November
Detection efficiency		Very high	High
Imagery tested		UAV (RGB+NIR; 5 cm); Pleiades 1B (MSS; 50 cm); color orthophoto (RGB; 25 cm)	UAV (RGB+NIR; 5 cm and 50 cm); Pleiades 1B (MSS; 50 cm)
Data resolution required	Spatial	<50 cm	<50 cm
	Spectral	Low	Moderate (NIR)
	Temporal	Right timing important (1 month period)	Right timing important (1 month period)
Optimal approach		Object-based	Pixel-based

The giant hogweed (*H. mantegazzianum* Sommier and Levier, Apiaceae family), native to Caucasus Mountains, is an example of a species that is distinct from both the spectral and the structural point of view, with pronounced dynamics of seasonal development (flowering). It is the biggest herbaceous plant in Central Europe (2–5 m in height with leaves up to 2.5 m long), forming large white circular inflorescences of compound umbels (up to 80 cm wide; Page et al., 2006; Perglová et al., 2006). As a perennial monocarpic species it flowers once in a lifetime, mostly in the third year (Pergl et al., 2006); in the Czech Republic, the flowering season is from late June to early August (Perglová et al., 2006). The white inflorescences are distinct, but non-flowering plants are problematic to detect. Although, the species invades mostly unmanaged grasslands and anthropogenic habitats, and forms large monospecific populations, it also occurs at forest margins or in forest interiors where RS detection is difficult (Table 2). The same is true for grazed or mown plants, that survive as ground rosettes that do not flower or, if they do, it is late in the season and inflorescences are very small (Müllerová et al., 2013).

Knotweeds [*F. japonica* Houtt., *F. sachalinensis* (F. Schmidt) Nakai, and their hybrid *F*. × *bohemica* Chrtek and Chrtková] are example of species that are difficult to recognize by RS means because they form dense stands, relatively indistinct from similar vegetation types; the flowers are in white clustered racemes that appear from July to September in the Czech Republic. Morphological differences between the above-listed knotweeds are too subtle from the RS perspective to separate the two species and their hybrid. They are stout herbaceous perennials with robust erect stems up to 4 m tall and with highly heterogeneous canopy architecture. New leaves are dark red; stems and leaves turn orange/brown in late autumn. Although, knotweeds are considered as light-demanding species (Beerling et al., 1994; Bímová et al., 2003), they can grow under the forest canopy especially in areas disturbed by flooding, which makes the detection particularly difficult (Table 2). Still, the differences in phenology between knotweeds and the tree canopy (mainly the knotweed stem coloration late in the season after the canopy leaf abscission) could possibly allow for their detection using imagery from specific time or multitemporal, remotely-sensed imagery (cf. Shouse et al., 2013).

### Study area

**Figure 1:**
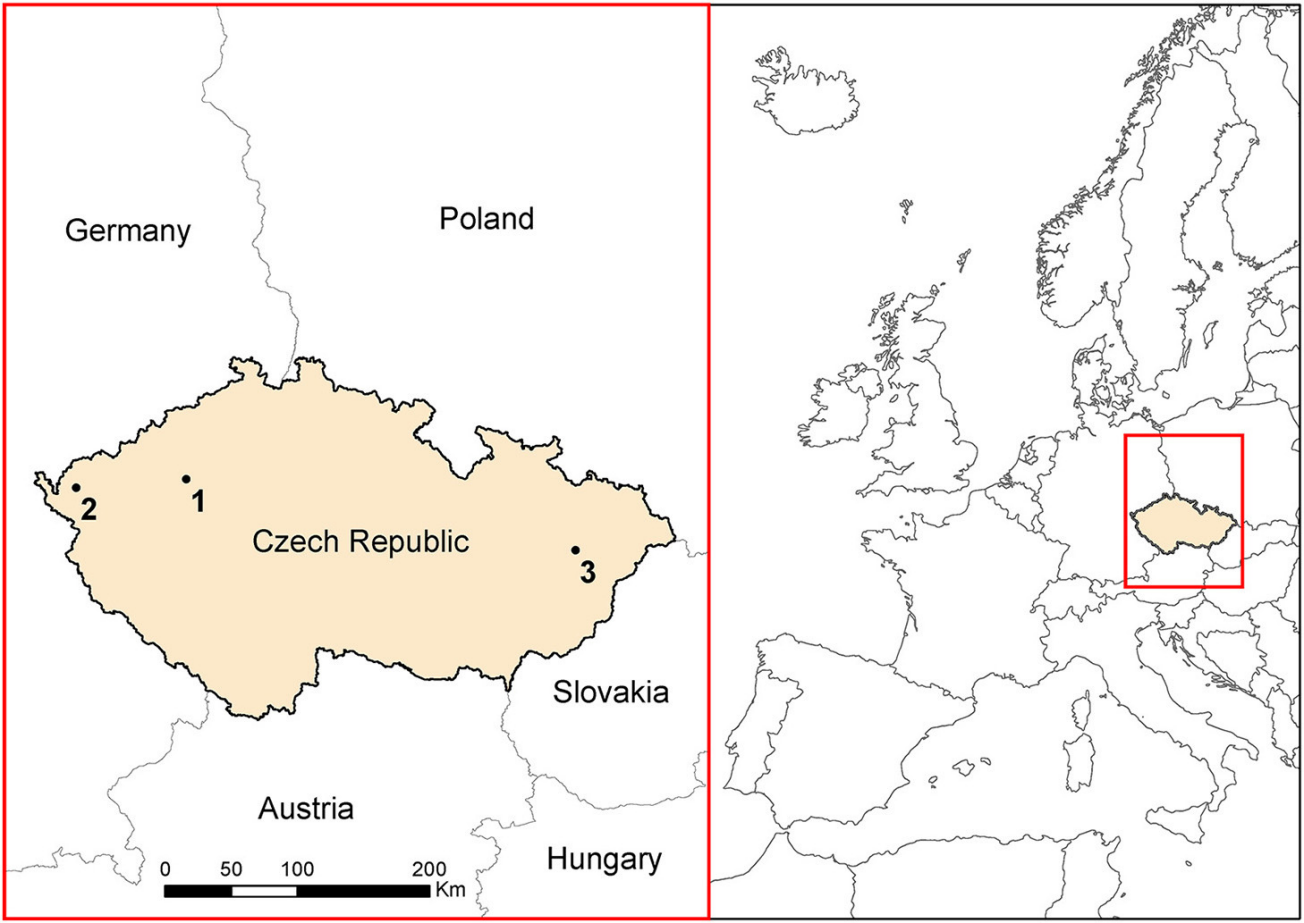
Study areas for both invasive species. Sites 1 and 2 account for giant hogweed, site 3 for knotweeds.

Three study sites were chosen in Czech Republic, Central Europe (Figure 1). For giant hogweed detection, these were: site 1 at a hilly landscape near Louny town (59 ha; 370 m a.s.l.), and site 2 in uplands near Sokolov town (70 ha; 600 m a.s.l.). Knotweeds were studied at site 3, situated in the lowland river floodplain around the Bečva river near Hranice na Moravě (96 ha; 250 m a. s. l.; Figure 1). RS data covered the variety of spectral, spatial, and temporal resolution (Table 3), such as the satellite imagery of Pleiades 1B (50 cm pan sharpened resolution; 4 channels; sites 1 and 3), color orthophoto (25 cm; site 1 and 2; CUZK, 2015), and UAV imagery (5 cm, RGB + NIR; all sites). For giant hogweed, we studied three phenological phases: the peak of flowering (beginning of July, site 2, UAV and aerial data), the end of flowering and the start of the fruit ripening (2nd half of July, site 1, all types of data), and post-flowering period (September, site 2, UAV, Table 3). For knotweeds, spring acquisition (May—UAV) captured early knotweed growth when many trees did not yet have leaves, summer one (July—UAV, August—satellite) captured the top of the vegetation season, and the autumn- (September—satellite, October—UAV) and late autumn acquisitions (November—UAV) recorded orange/red decaying knotweeds while most of the tree species were again without leaves. Availability of satellite imagery from the dates of the UAV acquisition was limited by cloudy weather.

**Table 3:** Overview of study sites and imagery used.

**Site No**.	**Name**	**Species**	**Area (ha)**	**Latitude**	**Longitude**	**Acquisition date**			**Phenological stage**
						**UAV**	**Satellite**	**Aerial**	
1.	Domoušice	*H. mantegazzianum*	59	50.23404	13.71937	15.7.2015	17.7.2015	17.7.2013	End of flowering
						20.7.2016			Ripening
2.	Anenská Ves	*H. mantegazzianum*	70	50.20937	12.53582	4.9.2014	–	1.7.2015	Peak of flowering
						9.7.2016			Out of bloom
3.	Skalička	*Fallopia* sp.	96	49.5318	17.7944	27.8.2015	7.7.2015	–	First leaves
						20.10.2015	9.9.2016		Top of vegetation season
							25.5.2016		Early senescence
							8.11.2016		Late senescence

*Gaps in the UAV imagery dataset are due to the technical problems with UAV*.

### Unmanned aircraft system deployed

For the purpose of this study, we designed a low-cost UAV capable of performing fully-automated mapping missions controlled by the APM ArduPlane/PixHawk autopilot. Its aerial segment is propelled by an electric BLDC motor, features a flying wing design and carries two modified consumer cameras (Sony Alpha A5100 with APS-C sensor and Sony E 20/2.8 lens). The first camera acquired standard RGB, and the second was modified to be sensitive in the NIR band by the removal of the built-in IR-cut filter and the addition of a Hoya R72 filter. This setup was used as a low-cost alternative to a more expensive multispectral camera (for more details on the UAV description see Müllerová et al., 2017). Using this UAV, we could test a suitable period in the seasonal development of the target species, focusing on times when it is possibly distinct from the background, such as, flowering for giant hogweed and autumn senescence for knotweeds.

### Image processing

#### Pre-processing

During the UAV flight mission, the sensors were periodically triggered with an overlap and sidelap ranging between 80 and 85% of the image height and width, respectively. This setting assured a robust mosaicking. Georeferencing was carried out using structure-from-motion approach (SfM; Dandois and Ellis, 2010; Westoby et al., 2012) in Agisoft PhotoScan Professional (Agisoft, 2016). This approach identifies similar features in conjugate images, tolerating large variations in scale and image acquisition geometry, generating very dense and accurate three-dimensional point clouds (Whitehead and Hugenholtz, 2014). Since the data are often linked to other field or remote sensing data either as a reference or for change detection, very high spatial resolution of UAV data implies the need for extremely accurate georeferencing. To assure high georeferencing accuracy, a GPS module capable of RAW data output (u-blox M8T) was connected to the autopilot. Triggering the two cameras was performed by the autopilot based on the distance traveled between two consecutive images (for more details see Müllerová et al., 2017).

Imagery from Pleiades 1B satellite was orthorectified using the Rational Polynomial Coefficients metadata provided and a digital surface model, and pan-sharpened to 0.5 m resolution. All imagery was afterwards visually checked and if necessary orthorectified using a national aerial orthophoto (CUZK, 2015) to ensure that the training areas and validation points cover the same areas in all UAV and satellite data.

#### Classification algorithms

Considering the inconsistent results in the comparison of different approaches to image classification (see e.g., Duro et al., 2012), we decided to test a large variety of algorithms (both pixel and object based) on both species and all three study sites. Whereas, the pixel-based approach uses only spectral information, the object-based approach takes into account also the spatial structure and context information. This approach is expected to improve the results by reducing the effects of shadows, within-class spectral variation and mis-registration, and is especially beneficial for detecting targets that take specific shape/form (Blaschke, 2010; Blaschke et al., 2014), such as giant hogweed inflorescences. It is also expected to be suitable for very high spatial resolution imagery (Laliberte and Rango, 2009). For the pixel-based classification, maximum likelihood (ML), a baseline classification method with equal probability of class assignment, and machine learning algorithms Support Vector Machine (SVM; Vapnik, 1995) and Random Forests (RF; Breiman, 2001) were employed in an ArcGIS 10.4.1 environment. Machine learning algorithms are assumed to be less sensitive to imbalanced training data sets (often the case in invasive ecology) since no assumptions are made about the distribution of input variables (Atkinson and Tatnall, 1997; Masocha and Skidmore, 2011). SVM is supposed be less susceptible to noise and correlated bands, especially useful for UAV pseudo NIR bands, and to better detect subtle and non-linear patterns (Foody and Mathur, 2004) whereas, RF classifier combining multiple classification trees (Breiman, 2001) is thought to be resistant to overfitting, strong interactions among the variables and small perturbations of the data (Pal, 2005; Cutler et al., 2007). For the object-based approach, several types of segmentation (multiresolution and contrast split) and classification (rule-based, SVM, and RF) were tested in eCognition Developer software. Multiresolution segmentation consecutively merges pixels or existing image objects minimizing the heterogeneity, whereas the contrast split segmentation divides the image into dark and bright objects based on the threshold, maximizing the contrast by an edge ratio algorithm (eCognition Developer 9.2 Reference Book, 2016). The parameters used to classify segmented objects in an iterative, rule-based classification were length/width ratio, maximum object size, brightness, mean channel value, circular standard deviation and layer mean, maximum pixel values, hue, saturation, and intensity transformation, and mean difference to darker neighbors. To train the machine learning algorithms, the following object features were used: mean, maximum, and minimum layer values and their standard deviation—both normal and circular, contrast to neighboring pixels, the geometry of objects such as extent and shape (area, border length, length/width ratio, asymmetry, compactness), textural measures (Haralick and Shanmugam, 1973) based on spatial relationships of pixels in a gray-level co-occurrence matrix, such as GLCM homogeneity, contrast, dissimilarity, and entropy. For the giant hogweed, we also tested the template matching function in eCognition, where objects are detected based on prior created “templates” generated from the imagery.

To separate the role of spectral and spatial resolution, coarser spatial resolution similar to Pleiades 1B satellite was simulated by resampling UAV data to 50 cm resolution and performing the same classification algorithms (in case of knotweeds). The same training areas were used for all classifiers (inspected visually and in the field). To account for knotweeds hidden within the canopy, knotweed validation points were stratified to 50 in open areas and 50 among the canopy or in shadows, although still visible on all imagery. This allowed us to disentangle the role of the canopy in determining success in classification at different parts of vegetation season.

### Field data and accuracy assessment

In case of extremely high spatial resolution of the UAV imagery, geometrical precision of the field data can be problematic. Differential GNSS with centimeter precision is costly and time-consuming, especially for larger scales. Moreover, in some cases, such as a forested environment or with complex geomorphology, the estimated precision drops to several decimeters or even meters. To overcome the problem we used an Android-based application Collector for ArcGIS (ESRI, 2016) installed on a tablet with an integrated GPS. This enabled manual delineation of patches of invasive species using custom base maps, such as UAV imagery or other available high resolution orthophotos, increasing the precision of collected data. Field data collected in 2015 and 2016 were divided into the training and validation part to make the two sets independent. The reflectance spectra of the target invasive and co-occurring species were collected at midday with a portable spectrometer, Spectral Evolution RS-3500 (spectral range 350–2500 nm) using a pistol grip and calibrated, using a 99% white reference panel. Spectra were averaged from 10 measurements, repeated 10 times per species at each site and at the same distance apart, where possible.

Validation was performed using 200 randomly-distributed points (stratified sampling, 50% within the target species polygons delineated in the field) outside the training areas. For this purpose, we merged all classes without the target species into one class (“background”). All validation points were visually inspected on all images to ensure they cover the target species. In cases where the land cover changed, the points were removed and new random points were generated. Some training areas had to be reduced to cover the species on all images, because the extent of the target species changed between the acquisitions. For accuracy, assessment and comparison of the role of imagery timing and resolution, user's (UA) and producer's accuracy (PA) were used (Congalton and Green, 1999; Foody, 2002).

## Results

**Figure 2:**
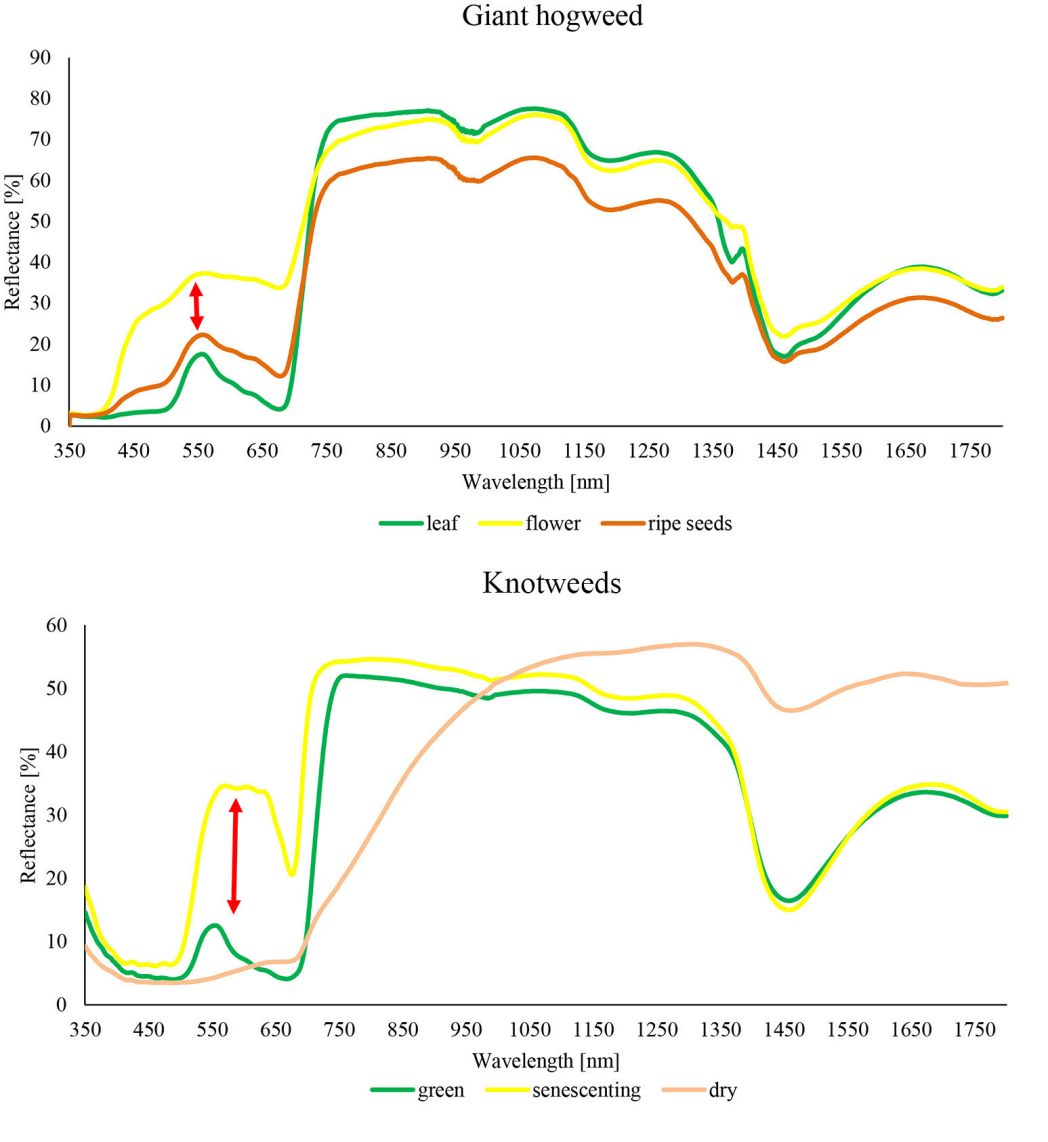
Reflectance curves of target species *H. mantegazzianum* and *F. japonica* measured by portable spectrometer Spectral Evolution RS-3500.

Our results show that both the detection accuracy and the best classification approach depend strongly on the phenological stage of plants, and on the spectral resolution of data (Table 2). This was true for both model species regardless of their markedly different phenological, structural and spectral characteristics, and seasonal dynamics. While giant hogweed was best detected when plants were flowering, thanks to its large white inflorescences, knotweeds were distinct at the phase of senescence because of reddish-brown coloring of decaying plants (Figure 2). In other parts of the season, detection of any of the species was more problematic and higher spectral resolution of satellite imagery improved the detection. The object-based approach was successful for giant hogweed if (i) the species was sampled in the right phenological stage and (ii) the spatial resolution enabled the distinction of individual compound umbels. For less distinct knotweeds spectral resolution of the data and pixel-based approach played more important role.

**Figure 3:**
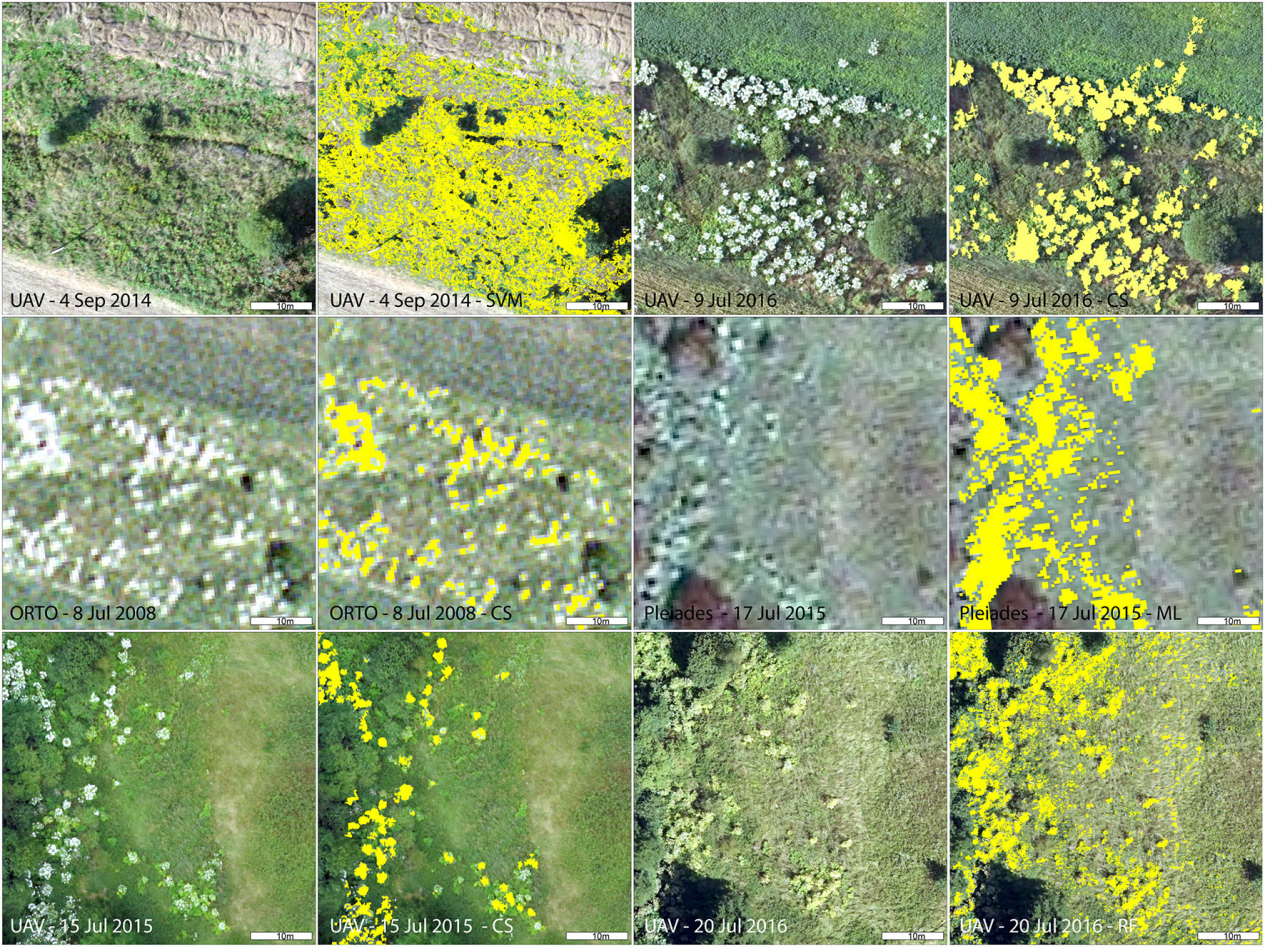
Detail of giant hogweed at different phenophases captured by various imagery (UAV, aerial, and Pleiades) and results of the best performing classification for each imagery. CS, contrast split segmentation; ML, Maximum Likelihood; RF, Random Forests object-based.

Giant hogweed was detected with a very high accuracy when flowering (up to 100%), dropping down later in the vegetation season to ~60% (September; Table 4). Flowering, undamaged individuals were detected with high accuracy, whereas non-flowering, fruiting, cut, sprayed, or grazed ones were more difficult to recognize due to their similarity with the surrounding vegetation. Further, the leaves surrounding flowering umbels were difficult to map (Figures 2, 3). The best results were obtained for full flowering (UAV imagery; 9 July; site 2) using an object-based approach—contrast split segmentation followed by rule-based classification (Figure 3). Contrast split segmentation itself separated very well the white objects on the imagery, meaning that all flowering hogweeds were detected. They were, however, mixed with other bright objects such as some artificial surfaces or harvested fields and had to be filtered out by further rule-based classification. At peak flowering, the inflorescences were so distinct that they could be detected with reasonable success (42/100% PA/UA of the hogweed class) even from a low spectral resolution aerial orthophoto (25 cm) using the same approach. However, the template-matching approach incorporated in eCognition software failed to recognize the umbels even at the peak of flowering. Depending on the threshold, it either picked all the hogweed umbels and many other objects of similar shape, even of different color or, if the threshold was lower, it missed half of the plants. It could work well if the infested area was previously masked out and the algorithm ran with high threshold only inside the infested polygons. This approach would require substantial manual input and would, thus, not be feasible to apply over larger datasets. In case of site 1, the summer UAV imagery was obtained later in the season (15 and 20 July) and part of the umbels in hogweed compound inflorescences had already started to ripen, slightly decreasing the accuracy. The contrast split rule-based approach of 15 July 2015 was the most effective, but failed completely for the imagery from 20 July 2016, when hogweed was mostly out of bloom. In the case of ripening hogweed (site 1), pixel-based approach and ML algorithm of the satellite imagery provided higher accuracy measures when compared to those derived from UAV. Still the accuracies were lower compared to full flowering hogweed on UAV imagery from site 1. In autumn, the UAV imagery depicted only leaves and dry stems, and the accuracy was very low (Table 4, Figure 3). In this case, no distinct objects could be identified and the object-based approach failed.

Table 4Classification accuracies (in %) for giant hogweed (*Heracleum mantegazzianum*) for UAV data (RGB, modified NIR; 5 cm), color orthophotos (RGB; 25 cm), and Pleiades (MSS; 2 m pan sharpened to 50 cm).

**Table:** 

**Site 1**		**UAV**														**Pleiades**						**Ortho photo**
**Classification**		**Pixel-based**								**Object-based**						**Pixel-based**			**Object-based**			
		**ML**		**SVM**		**RF**		**MRS**		**CS**		**SVM**		**RF**		**ML**	**SVM**	**RF**	**CS**	**SVM**	**RF**	**CS**
Acquisition date		15-Jul-2015	20-Jul-2016	15-Jul-2015	20-Jul-2016	15-Jul-2015	20-Jul-2016	15-Jul-2015	20-Jul-2016	**15-Jul-2015**	20-Jul-2016	15-Jul-2015	20-Jul-2016	15-Jul-2015	20-Jul-2016	**17-Jul-2015**	17-Jul-2015	17-Jul-2015	17-Jul-2015	17-Jul-2015	17-Jul-2015	17-Jul-2013
Overall accuracy		78	71	76	72	75	72	**83**	65	**83**	Failed	65	71	76	75	**91**	85	89	72	63	85	79
Hogweed class	PA	59	55	64	55	64	59	66	31	66		**69**	49	64	58	**86**	79	86	46	25	70	57
	UA	94	80	83	83	82	80	99	94	**100**		17	89	83	88	**94**	90	91	96	100	99	94

**Table:** 

**Site 2**		**UAV**														**Ortho photo**
**Classification**		**Pixel-based**								**Object-based**						
		**ML**		**SVM**		**RF**		**MRS**		**CS**		**SVM**		**RF**		**CS**
Acquisition date		4-Sep-2014	9-Jul-2016	4-Sep-2014	9-Jul-2016	4-Sep-2014	9-Jul-2016	4-Sep-2014	9-Jul-2016	4-Sep-2014	**9-Jul-2016**	4-Sep-2014	9-Jul-2016	4-Sep-2014	9-Jul-2016	8-Jul-2008
Overall accuracy		58	83	61	54	60	84	Failed	82	Failed	**100**	Failed	86	Failed	84	71
Hogweed class	PA	44	66	45	66	44	69		64		**99**		72		75	42
	UA	61	99	66	99	64	99		100		**100**		100		90	100

*The best results are highlighted in bold. Object-based analysis did not work for imagery from later phenophases, such cases are marked as “failed.” CS, contrast split segmentation followed by rule based classification; ML, Maximum Likelihood; MRS, multiresolution segmentation followed by rule based classification; PA, producer's accuracy; RF, Random Forests; SVM, Support Vector Machines; UA, user's accuracy*.

**Figure 4:**
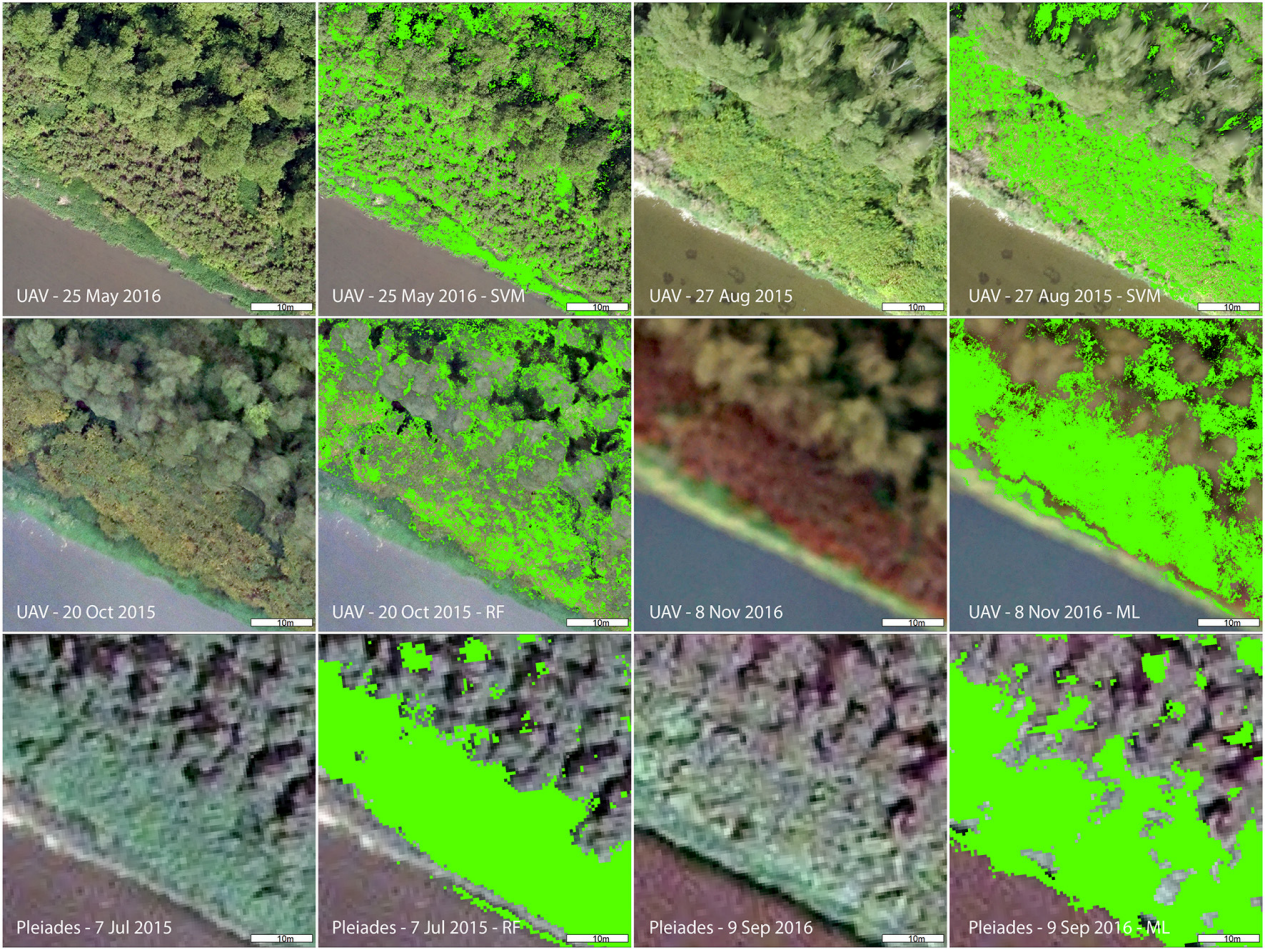
Detail of *Fallopia* sp. at different phenophases captured by various imagery (UAV and Pleiades) and results of the best performing classification per each imagery. ML, Maximum Likelihood; RF, Random Forest; SVM, Support Vector Machine.

The accuracies of knotweed classification varied considerably according to the phenology and spectral resolution (Table 5; Figure 4). The best results obtained were for the late-autumn UAV imagery (8 November; ML for both original and 50 cm resampled), when reddish-brown knotweeds stands were clearly visible (Figure 4). At other parts of the vegetation season, the PA derived from UAV imagery was low. Resampling of UAV data from 5 to 50 cm to simulate the Pleiades resolution slightly improved accuracies by eliminating salt and pepper effect, showing that spatial resolution does not limit species' recognition. Classification of summer Pleiades imagery using a RF classifier provided high accuracies (74/95% PA/UA of the knotweed class). Due to their architecture, knotweeds do not form objects of distinct shape or color, and object-based algorithms were therefore unsuccessful.

Table 5Classification accuracies (in %) for knotweed (*Fallopia* sp.) for UAV data (RGB, modified NIR; 5 cm, and resampled to 50 cm), and Pleiades (MSS; 2 m pan sharpened to 50 cm).

**Table:** 

**Classification**		**ML**				**SVM**				**RF**			
**Site 3**													
**UAV (5 cm)**													
Acquisition date		25-May-2016	27-Aug-2015	20-Oct-2015	8-Nov-2016	25-May-2016	27-Aug-2015	20-Oct-2015	8-Nov-2016	25-May-2016	27-Aug-2015	20-Oct-2015	8-Nov-2016
Overall accuracy		66	76	77	79	74	78	75	76	65	74	76	73
Knotweed	PA	44	53	63	80	55	60	57	54	37	57	67	47
Class	UA	77	77	86	78	87	92	88	95	82	86	82	96
**UAV RESAMPLED TO (50 cm)**													
Acquisition date		25-May-2016	27-Aug-2015	20-Oct-2015	8-Nov-2016	25-May-2016	27-Aug-2015	20-Oct-2015	8-Nov-2016	25-May-2016	27-Aug-2015	20-Oct-2015	8-Nov-2016
Overall accuracy		70	68	79	83	69	82	71	78	62	75	72	73
Knotweed	PA	47	43	74	82	47	67	51	60	31	55	56	50
class	UA	85	86	82	83	84	94	85	92	82	90	82	93

**Table:** 

**PLEIADES (2 M PAN SHARP. TO 50 cm)**							
**Classification**		**ML**		**SVM**		**RF**	
Acquisition date		7-Jul-2015	9-Sep-2016	7-Jul-2015	9-Sep-2016	7-Jul-2015	9-Sep-2016
Overall accuracy		80	72	85	70	85	66
Knotweed	PA	68	54	74	49	74	43
Class	UA	89	83	94	83	95	78

*The best results are highlighted in bold. Object-based analysis did not provide satisfactory results and is not shown. ML, Maximum Likelihood; PA, producer's accuracy; RF, Random Forests; SVM, Support Vector Machines; UA, user's accuracy*.

## Discussion

Our study addresses how different aspects of data quality (spatial, spectral, and temporal), costs, and operationality affect the methodology and practical application of the detection of invasive plants. Our two model species of different phenology and architecture, giant hogweed and knotweeds, showed different results reflecting their spectral characteristics and seasonal dynamics, still phenology played an important role in detection of both species (cf. Huang and Asner, 2009; Somodi et al., 2012; Müllerová et al., 2013; Bradley, 2014). The choice of the best data source (aerial color orthophoto, UAV with RGB + NIR modified consumer cameras, and Pleiades satellite MSS imagery) and classification approach (object or pixel based) depended strongly on the species' characteristics and their phenological stage.

We demonstrate that the spectral resolution of the low-cost solution, represented by an unmanned aircraft system, is limited (720–950 nm with variable response declining rapidly toward the longer wavelength; Müllerová et al., 2017), but that this limitation can to some extent be surpassed by identifying the right phenological stage when a more satisfactory detection rate is achievable even for the less distinct species such as knotweed. During the spring, when the knotweed stands are formed by the mixture of old red stems from the previous year and short emerging new regrowth stems, and summer acquisition, with dense, fully-developed indistinct stands, spectral resolution became more important for the detection. On the other hand, during the senescent phase the reddish color and better visibility of knotweeds under defoliated trees provided good detectability irrespective of the spectral quality. The problem with the canopy cover hiding knotweeds during the vegetation season was partly solved by using off-season imagery (from late autumn), but some individuals still remained hidden, covered by trunks or by their shadows.

The very fine spatial resolution of UAV imagery was not always beneficial. In case of hogweed, it actually overwhelmed the relevant spatial patterns and hampered the classification based on flowering objects since, in such a great detail, instead of the consistent white “dots” of hogweed inflorescences, individual umbels of each inflorescence were visible, eventually decreasing the classification accuracy (cf. Ustin and Santos, 2010). On the other hand, the fine detail enabled mapping of the hogweed leaves surrounding flowering umbels and, to some extent, non-flowering individuals. In case of knotweed (pixel-based approach), very fine spatial resolution of UAV data caused the salt and pepper effect, eliminated after resampling and being less pronounced in case of late autumn imagery and slightly blurred due to technical matters relating to cameras. Still, the resampling of summer UAV imagery lowered the success rate, probably because, in the forest, individual pixels of knotweeds visible among the canopy trees were merged into mixed pixels.

We achieved slightly higher giant hogweed detection rates compared to previous aerial photography analysis (Müllerová et al., 2013). Michez et al. (2016) used a similar approach, applying multiresolution segmentation (an object-based approach) and RF classifier on UAV imagery gaining higher overall accuracy; still since the authors do not provide the hogweed class accuracy, it is difficult to compare their results with ours. In contrast to our research, earlier studies of knotweed detection did not achieve the accuracies sufficient for operational application (Dorigo et al., 2012; Michez et al., 2016). Dorigo et al. (2012), who detected knotweed using a ratio of spring and summer aerial photography, applied a random-forest classifier based on pixels combined with textural information derived from a moving kernel. Jones et al. (2011) performed a rule-based object-oriented classification of aerial photography but provide no assessment of accuracy so their results are difficult to compare. Michez et al. (2016) applied multiresolution segmentation (an object-based approach) and RF classifier of UAV imagery, but provided only overall accuracies and Kappa index, and admitted that they did not reach sufficient accuracies for operational application. These authors show that the best results were provided by using very small objects (~30 cm in size) and spectral indices, which is actually similar to the pixel-based methods. In some cases, ML performed slightly better compared to the learning algorithms, still the differences were minor. The commonly accepted principle of machine learning algorithms outcompeting ML cannot therefore be taken as a rule of thumb (but see Andermann and Gloaguen, 2009; Otukei and Blaschke, 2010). For object-based classification, a rule-based algorithm was more successful compared to the machine learning, still the method is highly case-specific, less universal, and requires substantially more expert knowledge.

Trade-offs exist between spatial, spectral, and temporal resolution while minimizing cost and making an approach operationally viable (Wiens et al., 2009; Willis, 2015). The demands for both spatial and spectral resolution depend largely on the target plant species; good results can be achieved with substantially lower resolution if the vegetation is sampled at the phenological phase when the species is at its most distinct. Our study shows that proper timing and high spatial resolution can to some extent compensate for the lower spectral resolution of a low-cost, unmanned aerial system, and help to decrease the error of omission. This is important for practical applications as it minimizes the number of missed plants (Müllerová et al., 2013). Multispectral or even hyperspectral UAV sensors available on the market would definitely improve the resulting accuracy; our low-cost solution of modified consumer cameras provides substantially lower spectral quality (Müllerová et al., 2017). We believe, however, that such instruments would make the resulting methodology too costly and complex to be puts into operation for nature conservation or land management.

UAV technology with its high flexibility and low costs can provide an appropriate sampling method at a high spatio-temporal scale. We do, however, need to be aware of several limits for application in the management of invasive plants. Among these are legal constraints in those urban areas that are typically invaded by alien plants and under the focus of land managers (Pyšek and Hulme, 2005). UAV is well-suited for experimental studies, targeted monitoring and eradication control, yet the satellite imagery (if available at right phenological phases) provides more appropriate solution for larger areas. For species with distinct appearance aerial imagery should also be considered.

## Conclusions and lessons learned

The variety of data and especially flexibility of the UAV approach enabled us to assess the effect of timing of the data acquisition (i.e., the phenological stage of the target invasive species) and both spectral and spatial resolution on the detection success. Our research establishes a methodology for targeted timely monitoring of invasive species, provides land managers and nature protection with information on spatial distribution of invaders and serves as a baseline for assessment and modeling of spatial patterns and future spread of invasive plants. Nevertheless, it is necessary to be aware of limits of the RS detection, for example, if the target plant is under dense tree canopies or heavily damaged by mowing, grazing, or spraying. Our results indicate that the choice of the best classification method is case-specific, depending largely on both the target plant and imagery characteristics. The results from one methodological comparison should not, therefore, be mechanically transposed to other target species and data types. A detailed methodological analysis and testing of possible approaches such as ours is therefore a necessary step in finding optimal mapping strategies. For practical implementation of the proposed monitoring approach, the recommended time window should be wide enough to allow for organizing a flight campaign, and highly-flexible, small UAVs are a great choice, providing an opportunity to carry out the mission at the right time and in a cost-effective manner.

## Author contributions

JM contributed substantially to the conception of the work, data acquisition, processing, analysis and interpretation, and composing the manuscript. JB contributed substantially to the data acquisition, processing, analysis and interpretation, and composing the manuscript. TB contributed to the data acquisition and processing, and composing the manuscript. PD contributed to the data acquisition and processing, and composing the manuscript. PP contributed to the conception of the work and composing the manuscript. MV contributed to the field data acquisition, interpretation, and composing the manuscript.

### Conflict of interest statement

The authors declare that the research was conducted in the absence of any commercial or financial relationships that could be construed as a potential conflict of interest.

## Abbreviations

GNSS: Global Navigation Satellite System
PA: producer's accuracy
RF: random forests
RS: remote sensing
SVM: support vector machine
UA: user's accuracy
VHR: very high resolution.
